# Supplementary material for: Crossing exceptional points in non-Hermitian quantum systems
Source: Sci Adv. 2025 Jan 8;11(2):eadr8275. doi: 10.1126/sciadv.adr8275 (PMC11708895; doi:10.1126/sciadv.adr8275)
Supplement: Supplementary file 1 — Supplementary Text References [file sciadv.adr8275_sm.pdf]

Science Advances
AAAS

Supplementary Materials for

Crossing exceptional points in non-Hermitian quantum systems

Friederike U. J. Klauck *et al.*

Corresponding author: Tom A. W. Wolterink, tom.wolterink@uni-rostock.de

Sci. Adv. **11**, eadr8275 (2025)
DOI: 10.1126/sciadv.adr8275

This PDF file includes:

Supplementary Text
References

Supplementary Text

Hamiltonian of the sandwiched coupler

The lossy directional coupler is sandwiched between two 50/50 couplers, acting as rotation matrices. The time evolution can be described by

$$U_{sandwich} = R\,UR^{-1} = Re^{iHz}R^{-1} = e^{iRHR^{-1}z} = e^{iH_{sandwich}z}$$

The Hamiltonian of the sandwiched coupler is therefore given as:

$$H_{sandwich} = R\,HR^{-1}$$
$$= \frac{1}{\sqrt{2}}\begin{pmatrix} 1 & -i \\ -i & 1 \end{pmatrix}\begin{pmatrix} 0 & \kappa \\ \kappa & -2\,i\gamma \end{pmatrix}\frac{1}{\sqrt{2}}\begin{pmatrix} 1 & i \\ i & 1 \end{pmatrix}$$
$$= \frac{1}{2}\begin{pmatrix} 1 & -i \\ -i & 1 \end{pmatrix}\begin{pmatrix} i\kappa & \kappa \\ \kappa + 2\gamma & i\kappa - 2i\gamma \end{pmatrix}$$
$$= \begin{pmatrix} -i\gamma & \kappa - \gamma \\ \kappa + \gamma & -i\gamma \end{pmatrix}$$

PT-symmetry of the lossy couplers

The Hamiltonian H of a lossy directional coupler obeys passive PT symmetry. For all further purposes it is sufficient to consider the non-damped system with exact PT-symmetry that shows exactly the same dynamics (36). With T performing a complex conjugation and $P = \begin{pmatrix} 0 & 1 \\ 1 & 0 \end{pmatrix}$ the parity operator, the PT-symmetry condition can be written as

$$H = PH^*\,P^{-1}.$$

The Hamiltonian of the sandwiched coupler does not obey the same PT symmetry as the isolated coupler. However, to check if H_s fulfills any kind PT-symmetry, it has to commute with an antilinear operator (49). In our case, this means also transforming the parity operator in a similar way as our Hamiltonian.

The sandwiched Hamiltonian is obtained by rotating H:

$$H_S = R\,H\,R^{-1}$$
$$H_S = R\,(PH^*P^{-1})\,R^{-1}$$

Inserting the definition of the sandwiched Hamiltonian back in here gives

$$H_S = R\,P(R^{-1}\,H_S\,R)^*P^{-1}\,R^{-1}$$
$$H_S = R\,P(R^{-1})^*\,H_S^*\,R^*P^{-1}\,R^{-1}$$
$$H_S = H_S^*$$
$$H_S = P_s H_s^*\,{P_s}^{-1}$$

The parity operator of the original system transforms into the identity matrix. This means that the sandwiched Hamiltonian is then also PT-symmetric in the rotated basis, with the identity matrix as its parity operator P_s.

To move back to the passive PT-symmetric system, H_s transforms to $H_{sandwich} = H_s - i\gamma I$.

HOM dip visibilities

The visibility of the HOM dip characterizes the difference in coincidence rates for distinguishable and indistinguishable photons that travelled through a beam splitter. It is given by

$$vis = \frac{P_{indist}}{P_{dist}} - 1$$

with P_{indist} the probability of finding one photon in each of outputs of the beam splitter for an indistinguishable photon input and P_{dist} for a distinguishable input. Using the beam splitter's transmission matrix U, these can be expressed as:

$$P_{indist} = |U_{11}U_{22} + U_{12}U_{21}|^2$$
$$P_{dist} = |U_{11}U_{22}|^2 + |U_{12}U_{21}|^2$$

Clearly,

$$P_{indist} = P_{dist} + 2\,\mathrm{Re}(U_{11}U_{22}U_{12}{}^{*}U_{21}{}^{*})$$

And therefore the visibility can be rewritten as

$$vis = \frac{P_{dist}}{P_{dist}} + \frac{2\,\mathrm{Re}(U_{11}U_{22}U_{12}{}^{*}U_{21}{}^{*})}{|U_{11}U_{22}|^2 + |U_{12}U_{21}|^2} - 1$$
$$vis = \frac{2\,\mathrm{Re}(U_{11}U_{22}U_{12}{}^{*}U_{21}{}^{*})}{|U_{11}U_{22}|^2 + |U_{12}U_{21}|^2}$$

For the sandwiched coupler, the Hamiltonian of the system at the exceptional point, where $\kappa = \gamma$, becomes

$$H_{SEP} = \begin{pmatrix} -i\kappa & 0 \\ 2\kappa & -i\kappa \end{pmatrix}$$

Since ${H_{SEP}}_{12}$is zero, meaning there is no coupling of light from waveguide 2 to waveguide 1, also the term U_{12} in the transmission matrix of the system becomes zero. Inserting this into our formula for the visibility yields

$$vis = \frac{2\,\mathrm{Re}(U_{11}U_{22}U_{12}{}^{*}0)}{|U_{11}U_{22}|^2 + |U_{12}0|^2} = 0.$$

At the exceptional point, the probability of finding one photon in each waveguide is equal for distinguishable and indistinguishable photons, resulting in a HOM dip with zero visibility.

REFERENCES AND NOTES

1. T. Kato, “Perturbation theory in a finite-dimensional space” in *Perturbation Theory for Linear Operators*, T. Kato, Ed. (Springer, Berlin, Heidelberg, 1966), pp. 62–126.

2. R. El-Ganainy, K. G. Makris, M. Khajavikhan, Z. H. Musslimani, S. Rotter, D. N. Christodoulides, Non-Hermitian physics and PT symmetry. *Nat. Phys.* **14**, 11–19 (2018).

3. N. Moiseyev, P. R. Certain, F. Weinhold, Resonance properties of complex-rotated Hamiltonians. *Mol. Phys.* **36**, 1613–1630 (1978).

4. C. M. Bender, S. Boettcher, Real spectra in non-Hermitian hamiltonians having PT symmetry. *Phys. Rev. Lett.* **80**, 5243–5246 (1998).

5. K. G. Makris, R. El-Ganainy, D. N. Christodoulides, Z. H. Musslimani, Beam dynamics in P T symmetric optical lattices. *Phys. Rev. Lett.* **100**, 103904 (2008).

6. L. Feng, R. El-Ganainy, L. Ge, Non-Hermitian photonics based on parity–time symmetry. *Nat. Photonics* **11**, 752–762 (2017).

7. A. Li, H. Wei, M. Cotrufo, W. Chen, S. Mann, X. Ni, B. Xu, J. Chen, J. Wang, S. Fan, C.-W. Qiu, A. Alù, L. Chen, Exceptional points and non-Hermitian photonics at the nanoscale. *Nat. Nanotechnol.* **18**, 706–720 (2023).

8. C. Dembowski, H.-D. Gräf, H. L. Harney, A. Heine, W. D. Heiss, H. Rehfeld, A. Richter, Experimental observation of the topological structure of exceptional points. *Phys. Rev. Lett.* **86**, 787–790 (2001).

9. A. Guo, G. J. Salamo, D. Duchesne, R. Morandotti, M. Volatier-Ravat, V. Aimez, G. A. Siviloglou, D. N. Christodoulides, Observation of PT-symmetry breaking in complex optical potentials. *Phys. Rev. Lett.* **103**, 093902 (2009).

10. A. V. Sadovnikov, A. A. Zyablovsky, A. V. Dorofeenko, S. A. Nikitov, Exceptional-point phase transition in coupled magnonic waveguides. *Phys. Rev. Appl.* **18**, 024073 (2022).

11. Q. Zhong, R. El-Ganainy, Crossing exceptional points without phase transition. *Sci. Rep.* **9**, 134 (2019).

12. C. E. Rüter, K. G. Makris, R. El-Ganainy, D. N. Christodoulides, M. Segev, D. Kip, Observation of parity-time symmetry in optics. *Nat. Phys.* **6**, 192–195 (2010).

13. J. Doppler, A. A. Mailybaev, J. Böhm, U. Kuhl, A. Girschik, F. Libisch, T. J. Milburn, P. Rabl, N. Moiseyev, S. Rotter, Dynamically encircling an exceptional point for asymmetric mode switching. *Nature* **537**, 76–79 (2016).

14. C. Guria, Q. Zhong, S. K. Ozdemir, Y. S. S. Patil, R. El-Ganainy, J. G. E. Harris, Resolving the topology of encircling multiple exceptional points. *Nat. Commun.* **15**, 1369 (2024).

15. H. Hodaei, A. U. Hassan, S. Wittek, H. Garcia-Gracia, R. El-Ganainy, D. N. Christodoulides, M. Khajavikhan, Enhanced sensitivity at higher-order exceptional points. *Nature* **548**, 187–191 (2017).

16. W. Chen, Ş. Kaya Özdemir, G. Zhao, J. Wiersig, L. Yang, Exceptional points enhance sensing in an optical microcavity. *Nature* **548**, 192–196 (2017).

17. H. Hodaei, M.-A. Miri, M. Heinrich, D. N. Christodoulides, M. Khajavikhan, Parity-time–symmetric microring lasers. *Science* **346**, 975–978 (2014).

18. L. Feng, Z. J. Wong, R.-M. Ma, Y. Wang, X. Zhang, Single-mode laser by parity-time symmetry breaking. *Science* **346**, 972–975 (2014).

19. H. Defienne, M. Barbieri, I. A. Walmsley, B. J. Smith, S. Gigan, Two-photon quantum walk in a multimode fiber. *Sci. Adv.* **2**, e1501054 (2016).

20. T. A. W. Wolterink, R. Uppu, G. Ctistis, W. L. Vos, K.-J. Boller, P. W. H. Pinkse, Programmable two-photon quantum interference in 10^3 channels in opaque scattering media. *Phys. Rev. A* **93**, 053817 (2016).

21. B. Vest, M.-C. Dheur, É. Devaux, A. Baron, E. Rousseau, J.-P. Hugonin, J.-J. Greffet, G. Messin, F. Marquier, Anti-coalescence of bosons on a lossy beam splitter. *Science* **356**, 1373–1376 (2017).

22. L. Hong, Y. Zhang, Y. Chen, L. Chen, Loss-assisted anomalous Hong-Ou-Mandel interference based on nonunitary multilayer graphene. *Phys. Rev. Lett.* **133**, 023601 (2024).

23. S. M. Barnett, J. Jeffers, A. Gatti, R. Loudon, Quantum optics of lossy beam splitters. *Phys. Rev. A* **57**, 2134–2145 (1998).

24. R. Uppu, T. A. W. Wolterink, T. B. H. Tentrup, P. W. H. Pinkse, Quantum optics of lossy asymmetric beam splitters. *Opt. Express* **24**, 16440–16449 (2016).

25. J. Huber, P. Kirton, S. Rotter, P. Rabl, Emergence of PT-symmetry breaking in open quantum systems. *SciPost Phys.* **9**, 052 (2020).

26. M. Znojil, Passage through exceptional point: Case study. *Proc. R. Soc. Math. Phys. Eng. Sci.* **476**, 20190831 (2020).

27. W. Cao, X. Lu, X. Meng, J. Sun, H. Shen, Y. Xiao, Reservoir-mediated quantum correlations in non-hermitian optical system. *Phys. Rev. Lett.* **124**, 030401 (2020).

28. J. Zhou, Characterization of PT-symmetric quantum interference based on the coupled mode theory. *Opt. Express* **30**, 23600–23607 (2022).

29. C. A. Downing, A. Vidiella-Barranco, Parametrically driving a quantum oscillator into exceptionality. *Sci. Rep.* **13**, 11004 (2023).

30. F. Roccati, A. Purkayastha, G. M. Palma, F. Ciccarello, Quantum correlations in dissipative gain–loss systems across exceptional points. *Eur. Phys. J. Spec. Top.* **232**, 1783–1788 (2023).

31. F. Klauck, L. Teuber, M. Ornigotti, M. Heinrich, S. Scheel, A. Szameit, Observation of PT-symmetric quantum interference. *Nat. Photonics* **13**, 883–887 (2019).

32. N. Maraviglia, P. Yard, R. Wakefield, J. Carolan, C. Sparrow, L. Chakhmakhchyan, C. Harrold, T. Hashimoto, N. Matsuda, A. K. Harter, Y. N. Joglekar, A. Laing, Photonic quantum simulations of coupled PT-symmetric Hamiltonians. *Phys. Rev. Res.* **4**, 013051 (2022).

33. S. Longhi, Quantum statistical signature of PT symmetry breaking. *Opt. Lett.* **45**, 1591–1594 (2020).

34. T. A. W. Wolterink, M. Heinrich, S. Scheel, A. Szameit, Order-invariant two-photon quantum correlations in PT-symmetric interferometers. *ACS Photonics* **10**, 3451–3457 (2023).

35. C. K. Hong, Z. Y. Ou, L. Mandel, Measurement of subpicosecond time intervals between two photons by interference. *Phys. Rev. Lett.* **59**, 2044–2046 (1987).

36. M. Ornigotti, A. Szameit, Quasi PT-symmetry in passive photonic lattices. *J. Opt.* **16**, 065501 (2014).

37. S. Scheel, A. Szameit, PT -symmetric photonic quantum systems with gain and loss do not exist. *EPL Europhys. Lett.* **122**, 34001 (2018).

38. L. Teuber, S. Scheel, Solving the quantum master equation of coupled harmonic oscillators with Lie-algebra methods. *Phys. Rev. A* **101**, 042124 (2020).

39. M. Gräfe, R. Heilmann, R. Keil, T. Eichelkraut, M. Heinrich, S. Nolte, A. Szameit, Correlations of indistinguishable particles in non-Hermitian lattices. *New J. Phys.* **15**, 033008 (2013).

40. A. Szameit, F. Dreisow, T. Pertsch, S. Nolte, A. Tünnermann, Control of directional evanescent coupling in fs laser written waveguides. *Opt. Express* **15**, 1579–1587 (2007).

41. T. Eichelkraut, S. Weimann, S. Stützer, S. Nolte, A. Szameit, Radiation-loss management in modulated waveguides. *Opt. Lett.* **39**, 6831–6834 (2014).

42. M. Ehrhardt, M. Heinrich, A. Szameit, Observation-dependent suppression and enhancement of two-photon coincidences by tailored losses. *Nat. Photonics* **16**, 191–195 (2022).

43. S. Longhi, Quantum interference and exceptional points. *Opt. Lett.* **43**, 5371–5374 (2018).

44. B. Longstaff, E.-M. Graefe, Nonadiabatic transitions through exceptional points in the band structure of a PT-symmetric lattice. *Phys. Rev. A* **100**, 052119 (2019).

45. Z.-N. Tian, F. Yu, X.-L. Zhang, K. M. Lau, L.-C. Wang, J. Li, C. T. Chan, Q.-D. Chen, On-chip single-photon chirality encircling exceptional points. *Chip* **2**, 100066 (2023).

46. W. C. Wong, J. Li, Exceptional-point sensing with a quantum interferometer. *New J. Phys.* **25**, 033018 (2023).

47. K. M. Davis, K. Miura, N. Sugimoto, K. Hirao, Writing waveguides in glass with a femtosecond laser. *Opt. Lett.* **21**, 1729–1731 (1996).

48. S. Weimann, T. Eichelkraut, A. Szameit, Decay of bound states in oscillating potential wells. *Phys. Rev. A* **97**, 053844 (2018).

49. C. M. Bender, M. V. Berry, A. Mandilara, Generalized PT symmetry and real spectra. *J. Phys. A* **35** L467–L471 (2002).
